# Prescribing of long-acting beta-2-agonists/inhaled corticosteroids after the SMART trial

**DOI:** 10.1186/s12890-015-0051-x

**Published:** 2015-05-06

**Authors:** Marietta Rottenkolber, Rainald Fischer, Luisa Ibáñez, Joan Fortuny, Robert Reynolds, Justyna Amelio, Roman Gerlach, Martin Tauscher, Petra Thürmann, Joerg Hasford, Sven Schmiedl

**Affiliations:** Institute for Medical Information Sciences, Biometry, and Epidemiology, Ludwig-Maximilians-Universitaet, Marchioninistr. 15, D-81377 Munich, Germany; Pneumologische Praxis Muenchen – Pasing, Munich, Germany; Fundació Institut Català de Farmacologia, Hospital Universitari Vall d’Hebron, Barcelona, Spain; Departament de Farmacologia, Terapèutica i Toxicologia, Universitat Autònoma de Barcelona, Barcelona, Spain; Novartis Farmaceutica SA, Barcelona, Spain; Pfizer Research & Development, New York, USA; Amgen Ltd, Uxbridge, UK; National Association of Statutory Health Insurance Physicians of Bavaria, Munich, Germany; Department of Clinical Pharmacology, School of Medicine, Faculty of Health, Witten/Herdecke University, Witten, Germany; Philipp Klee-Institute for Clinical Pharmacology, HELIOS Clinic Wuppertal, Wuppertal, Germany

**Keywords:** Long-acting adrenergic beta-2-receptor agonists, Inhaled corticosteroids, Asthma, COPD, ACOS, Drug utilisation study, SMART trial, Drug regulatory actions

## Abstract

**Background:**

After the SMART trial evaluating the safety of salmeterol (long-acting beta-2-agonist (LABA)) in asthma patients, regulatory actions were taken to promote a guideline-adherent prescribing of LABA only to patients receiving inhaled corticosteroids (ICS). We aim to analyse LABA- and ICS-related prescription patterns after the SMART trial in Germany.

**Methods:**

Patients documented in the Bavarian Association of Statutory Health Insurance Physicians database (approximately 10.5 million people) were included if they had a diagnosis of asthma and at least one prescription of LABA and/or ICS between 2004 and 2008. Annual period prevalence rates (PPRs) were estimated and Cochrane Armitage tests were used for time trend analyses.

**Results:**

Highest annual PPRs were found for budesonide and the fixed combination of salmeterol/fluticasone. The proportion of “concomitant LABA and ICS users” increased from 52.0 to 57.6% within the study period, whereas for “LABA users without ICS” a slight decrease from 6.5 to 5.4% was found. In 2008, the proportion of patients with at least one quarter with a LABA prescription without concomitant ICS was highest in elderly, male patients (≈20%). In the majority of these patients, a concomitant diagnosis of COPD (i.e. asthma-COPD overlap syndrome [ACOS]) was present.

**Conclusions:**

Between 2004 and 2008, we found a moderate increase in guideline-adherent LABA prescribing in a representative German population. Elderly men received a significant number of LABA prescriptions without concomitant ICS probably due to ACOS.

**Electronic supplementary material:**

The online version of this article (doi:10.1186/s12890-015-0051-x) contains supplementary material, which is available to authorized users.

## Background

The Salmeterol Multicentre Asthma Research Trial (SMART) [1] was a large randomized controlled trial in asthma patients evaluating the safety of salmeterol (i.e., a long-acting beta-2-agonist [LABA]) compared to placebo in addition to usual asthma care. In 2003, this trial was prematurely terminated by GlaxoSmithKline due to recruitment problems and safety issues. In an interim analysis, a non-significant increase in combined respiratory-related deaths or life-threatening events was found in patients receiving salmeterol but for African Americans, this increase was statistically significant. Furthermore, a significantly increased risk for “combined respiratory-related death or life-threatening experience” (RR = 5.6, 95% CI: 1.2-25.3) and “combined asthma-related deaths or life-threatening experience” (RR = 10.5, 95% CI: 1.3-81.6) was found for African Americans who had no prescription of an inhaled corticosteroid (ICS) at baseline. In contrast, if ICS was present at baseline no significant differences between patients receiving salmeterol or placebo were found [1]. Regarding these results as well as previously and recently published guidelines, LABA should be prescribed only to patients receiving ICS [2,3].

In 2003, “Dear doctor” letters were sent out by GlaxoSmithKline and detailed results of the SMART trial were added to the respective SPCs of LABA-containing products [4-6]. After presentation of SMART results to the public, an intensive and somewhat controversial discussion occurred between the stakeholders [7]. In 2005, information for health care providers were sent out by the FDA stating that LABA should be prescribed to asthma patients only if other medicines, including low-or-medium dose ICS, do not control asthma [8-10] and a ‘Black box’ warning on LABA was imposed by the FDA [11,12]. In Germany, the national drug regulatory authority (“BfArM”) published in August 2003 a statement [13] presenting results of the SMART trial and pointing out the need for a guideline-adherent treatment [14]. In 2006, it was decided to add warnings similar to those made by the FDA to product labelling of LABA compounds in Germany, too [15].

Taking into account the essential need for a concomitant ICS usage in patients receiving LABA, fixed combination of LABA/ICS might be considered as a meaningful treatment option for patients with asthma and respective recommendations were made by the FDA [16,17] and by several guidelines [3,17,18].

Despite the importance of obtaining a reliable picture of real-life prescription behaviour after the SMART trial and related regulatory actions, only a few data exists analysing changes in LABA- and ICS-related prescribing in detail [19,20]. Hence, we aim to analyse trends in prescriptions of LABA, ICS, and fixed combination drugs containing LABA and ICS between 2004 and 2008 using a German database covering 10.5 million people.

## Methods

### Study type and data source

A drug utilization study was conducted in the database of the Association of Statutory Health Insurance Physicians, Bavaria [21]. This population-based database covers all compulsorily insured persons of the Statutory Health Insurance. The database has existed since 2001 and covers 85% (i.e., 10.5 million people) of the total Bavarian population excluding those with a private health insurance. It compiles, based on accounting information of Bavarian physicians, the patient characteristics, diagnoses of both general practitioners and consultants, all performed medical services, and drug utilization of all outpatients. Diagnoses and patient characteristics are documented on a patient-related basis. All information is updated quarterly; i.e., for each diagnosis or prescription the quarter is documented in the database, rather than the actual prescription date. Prescriptions are only recorded in the database if they are filled at the pharmacy. The International Statistical Classification of Diseases and Related Health Problems terminology (ICD-10-GM) was used for coding diagnoses and the Anatomical Therapeutic Chemical classification system (ATC) for coding drugs [22,23]. The study period included the years 2004–2008. All analyses were performed using anonymized administrative data only. Thus an ethical approval is not needed in Germany. There was neither a data protection nor a legal basis to ask for an ethical review or approval. The data of the National Association of Statutory Health Insurance Physicians of Bavaria, Munich, Germany (KVB) was routinely collected on a legal basis. All authors had direct access to KVB anonymized raw data for statistical analyses. For this project a written agreement was signed between the Institute of Medical Information Sciences, Biometry, and Epidemiology (LMU Muenchen) and the Bavarian National Association of Statutory Health Insurance Physicians. Finally, all analyses were performed based on STROBE (“STrengthening the Reporting of OBservational studies in Epidemiology”) guidelines and the researchers assured that data was handled properly and stored on secured servers.

### Patient population

Patients having at least one ICD-10-GM diagnostic code of asthma (J45 (“Asthma”) and/or J46 (“Status asthmaticus”)) documented within the study period and at least one prescription of the following respiratory drugs within the study period were included: salmeterol (ATC code: R03AC12), formoterol (R03AC13), salmeterol and fluticasone (R03AK06), formoterol and beclometasone (R03AK27), formoterol and budesonide (R03AK28), beclometasone (R03BA01), budesonide (R03BA02), flunisolide (R03BA03), betamethasone (R03BA04), fluticasone (R03BA05), triamcinolone (R03BA06), mometasone (R03BA07), and ciclesonide (R03BA08). The index date was set as the quarter of the year of the first prescription of a drug of interest in the study period. Patients with an additional diagnosis of COPD (ICD-10-GM: J44 [“Other chronic obstructive pulmonary disease”]) were considered as patients with asthma-COPD overlap syndrome (ACOS).

### Definition concomitant usage of LABA and ICS

If a patient received a fixed combination drug containing LABA and ICS, the respective quarter of the year was considered as a quarter with concomitant usage of LABA and ICS (irrespective of any other drugs). Concomitant usage of LABA and ICS could also be assured by prescribing both compounds separately, but in a close temporal relationship (usually at the same day in clinical routine). Taking into account a quarterly documentation of prescribed drugs, a separate prescription of both a LABA and an ICS compound was considered as concomitant usage of LABA and ICS if given in one quarter (irrespective of any other drugs). According to the pattern of LABA and ICS prescriptions, patients were assigned to the following five mutually exclusive categories: “concomitant LABA and ICS users”, “switchers”, “non-concomitant LABA and ICS users”, “LABA users without ICS”, and “ICS users without LABA” (Table 1). “Switchers” were defined as patients with at least one prescription of concomitant LABA and ICS (fixed dose or separate prescription) in at least one quarter and at least one LABA prescription without ICS in at least one other quarter within a particular calendar year.

**Table 1:** **Treatment categories**

	**At least one prescription in at least one quarter within a particular calendar year**		
**Category**	**LABA & ICS (fixed or non-fixed in the same quarter)**	**LABA (no ICS)**	**ICS (no LABA)**
Concomitant LABA/ICS users	X	-	Possible*
Switchers	X	X	Possible**
Non-concomitant LABA/ICS users	-	X	X**
LABA users without ICS	-	X	-
ICS users without LABA	-	-	X*

*in up to all quarters of a particular calendar year, **except from the quarters with a LABA only prescription in up to all quarters of a particular calendar year.

In a subgroup analysis, the following further stratification were performed for the category “concomitant LABA and ICS users”: i) patients receiving LABA and ICS only in fixed inhalers (“combined inhaler”), ii) patients receiving LABA and ICS only in separate inhalers (“separate inhalers”); iii) patients with combinations (i.e., patients with at least one prescription of a fixed LABA/ICS device, and in addition a separate ICS- or a non-fixed LABA/ICS-prescription in the same or another quarter of the respective calendar year; “combinations”). All assignments to treatment groups were made on a calendar year basis.

### Statistical analysis

Annual period prevalence rates (PPRs) were calculated using the number of patients with at least one prescription of interest during the year of interest (numerator) divided by the total number of compulsorily insured Bavarians at midyear of the year of interest (July, 1; denominator) [24]. Annual PPRs per 10,000 persons were calculated stratified by age (ten-year age groups [0–9 years, 10–19 years, 20–29 years, […], 90+ years]), sex, and compound. For the five patient categories (“concomitant LABA and ICS users”, “switchers”, “non-concomitant LABA and ICS users”, “LABA users without ICS”, and ”ICS users without LABA”) and the subgroup categories “combined inhalers”, “separate inhalers”, and “combinations” the number of patients and proportions were calculated and stratifications by age, sex, and calendar year were performed. All time trend analyses were performed using the Cochrane Armitage test. All statistical calculations were conducted using IBM SPSS Statistics Version 20.0 and GNU R Version 3.0.1 (http://www.r-project.org/).

## Results

### Period prevalence rates

**Figure 1:**
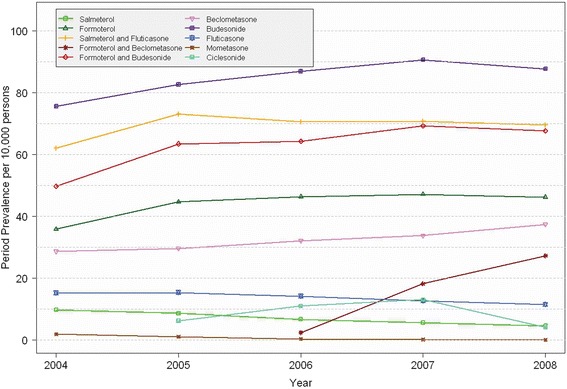
Annual period prevalence rates per 10,000 persons stratified by compound between 2004 and 2008.

Within the study period, the highest annual PPRs were found for budesonide (between 75.6 and 90.6 per 10,000 persons) and the fixed combination of salmeterol/fluticasone (between 62.1 and 73.1 per 10,000 persons). In contrast, the lowest PPRs were observed for mometasone (between 0.1 and 1.8 per 10,000 persons, Additional file 1: Table S1). From 2004 to 2008, a significant increase in PPRs was revealed for formoterol, fixed combinations of salmeterol/fluticasone, formoterol/beclometasone, formoterol/budesonide, and the ICS beclometasone and budesonide. For all remaining drugs including salmeterol, a decrease was found between 2004 and 2008 (all p-values <0.0001; Figure 1). In addition, a slight decrease was found for salmeterol/fluticasone (between 2005 and 2008), and for formoterol and formoterol/budesonide (between 2007 and 2008).

### Analysis of concomitant LABA and ICS usage

In total, 307,358 patients (approximately 2.9% out of all insured people) with a documented diagnosis of asthma or status asthmaticus were treated with at least one drug of interest in 2008. The highest proportion (57.6%) of patients was classified as “concomitant LABA and ICS users” followed by “ICS users without LABA” (31.4%, Table 2).

**Table 2:** **Age and sex distribution for the different treatment groups (“concomitant LABA and ICS users”, “switchers”, “non-concomitant LABA and ICS users”, “LABA users without ICS”, “ICS users without LABA”) for the year 2008**

	**Concomitant LABA and ICS users**	**Switchers**	**Non-concomitant LABA and ICS users**	**LABA users without ICS**	**ICS users without LABA**
**N (%)**	177,159 (57.6%)	14,899 (4.8%)	1,936 (0.6%)	16,749 (5.4%)	96,615 (31.4%)
**Age in years (mean ± SD)**	49.1 ± 21.9	59.1 ± 17.8	55.4 ± 20.2	58.0 ± 19.3	37.5 ± 24.9
**Females (n [%])**	98,071 (55.4%)	8,258 (55.4%)	1,184 (61.2%)	8,705 (52.0%)	52,907 (54.8%)

The proportion of asthma patients classified as “concomitant LABA and ICS users” increased from 52.0% (2004) to 57.6% (2008, p < 0.0001), whereas the proportion of patients classified as “LABA users without ICS” decreased from 2004 onwards (2004: 6.5%; 2008: 5.4%, p < 0.0001). The proportion of “switchers” decreased slightly during the study period (2004: 5.4%; 2008: 4.8%, p < 0.0001) and the proportion of “non-concomitant LABA and ICS users” increased slightly during the study period (2004: 0.55%; 2008: 0.63%, p = 0.0020). For patients classified as “ICS users without LABA”, we found a decrease within the study period (2004: 35.6%; 2008:31.4%, p < 0.0001, Table 3).

**Table 3:** **Proportion of patients stratified by treatment group (“concomitant LABA and ICS users”, “Switchers”, “non-concomitant LABA and ICS users”, “LABA users without ICS”, “ICS users without LABA”) for the years 2004 to 2008**

	**Year**				
	**2004**	**2005**	**2006**	**2007**	**2008**
**Concomitant LABA and ICS users**	123,873 (52.0%)	152,857 (54.6%)	152,418 (53.3%)	172,225 (55.3%)	177,159 (57.6%)
**Switchers**	12,785 (5.4%)	13,266 (4.7%)	14,207 (5.0%)	14,446 (4.6%)	14,899 (4.8%)
**Non-concomitant LABA and ICS users**	1,314 (0.6%)	1,686 (0.6%)	1,992 (0.7%)	1,896 (0.6%)	1,936 (0.6%)
**LABA users without ICS**	15,612 (6.5%)	19,200 (6.9%)	18,193 (6.4%)	17,810 (5.7%)	16,749 (5.4%)
**ICS users without LABA**	84,818 (35.6%)	92,970 (33.2%)	99,334 (34.7%)	105,252 (33.8%)	96,615 (31.4%)

The age and sex distribution (for the year 2008) for each treatment category is presented in Table 2. The mean age was the lowest in the “ICS users without LABA” group with 37.5 (standard deviation (SD): 24.9) years and the highest in the “switchers” group with 59.1 (SD: 17.8) years. In each group, more than half of all patients were females. In the “non-concomitant LABA and ICS users” group, the proportion of females was the highest (61.2%).

The proportion of patients with at least one LABA prescription without concomitant ICS (combined analysis of “LABA users without ICS”, “non-concomitant LABA and ICS users”, “switchers”) was the lowest (1.3%) in the age group 0–9 years, increased continuously over the age groups, peaked in patients aged 80–89 years (19.1%) and was followed by a small decrease for patients in the age group “90+” years (18.8%, Table 4). Regarding sex-related differences, the proportion of patients with at least one LABA prescription without concomitant ICS was slightly higher in men reaching more than 20% in men aged over 70 years (70–79 years: 20.3%, 80–89 years: 21.3%, “90+”: 21.4%; Table 4). In these elderly male patients, a concomitant diagnosis of COPD (i.e. asthma-COPD overlap syndrome) was present in 76.8% (Additional file 1: Table S2).

**Table 4:** Proportion of patients stratified by age group, sex, and treatment group (“concomitant LABA and ICS users”, “switchers”, “non-concomitant LABA and ICS users”, “LABA users without ICS”, “ICS users without LABA”) for the year 2008*

**Age group**	**Concomitant LABA and ICS users**		**Switchers**		**Non-concomitant LABA and ICS users**		**LABA users without ICS**		**ICS users without LABA**		**All LABA/ICS treatment categories**	
	**Male**	**Female**	**Male**	**Female**	**Male**	**Female**	**Male**	**Female**	**Male**	**Female**	**Male**	**Female**
**0-9**	4,242 (25.2%)	2,288 (24.2%)	103 (0.6%)	53 (0.6%)	36 (0.2%)	20 (0.2%)	95 (0.6%)	32 (0.3%)	12,376 (73.4%)	7,053 (74.7%)	16,852 (100%)	9,446 (100%)
**10-19**	10,347 (56.9%)	7,060 (56.1%)	247 (1.4%)	151 (1.2%)	60 (0.3%)	47 (0.4%)	434 (2.4%)	294 (2.3%)	7,095 (39.0%)	5,035 (40.0%)	18,183 (100%)	12,587 (100%)
**20-29**	6,660 (63.6%)	8,014 (60.8%)	214 (2.0%)	329 (2.5%)	23 (0.2%)	48 (0.4%)	448 (4.3%)	401 (3.0%)	3,123 (29.8%)	4,385 (33.3%)	10,468 (100%)	13,177 (100%)
**30-39**	7,972 (62.5%)	10,363 (58.7%)	386 (3.0%)	585 (3.3%)	54 (0.4%)	101 (0.6%)	579 (4.5%)	698 (4.0%)	3,758 (29.5%)	5,914 (33.5%)	12,749 (100%)	17,661 (100%)
**40-49**	11,503 (63.2%)	16,413 (60.4%)	790 (4.3%)	1,129 (4.2%)	95 (0.5%)	179 (0.7%)	904 (5.0%)	1,224 (4.5%)	4,916 (27.0%)	8,222 (30.3%)	18,208 (100%)	27,167 (100%)
**50-59**	10,310 (62.4%)	15,611 (60.0%)	1,000 (6.1%)	1,494 (5.7%)	112 (0.7%)	209 (0.8%)	1,126 (6.8%)	1,436 (5.5%)	3,962 (24.0%)	7,261 (27.9%)	16,510 (100%)	26,011 (100%)
**60-69**	12,488 (61.6%)	16,885 (59.7%)	1,659 (8.2%)	1,921 (6.8%)	148 (0.7%)	242 (0.9%)	1,779 (8.8%)	1,794 (6.3%)	4,208 (20.7%)	7,464 (26.4%)	20,282 (100%)	28,306 (100%)
**70-79**	11,291 (61.7%)	14,340 (60.9%)	1,673 (9.1%)	1,780 (7.6%)	180 (1.0%)	229 (1.0%)	1,868 (10.2%)	1,770 (7.5%)	3,294 (18.0%)	5,445 (23.1%)	18,306 (100%)	23,564 (100%)
**80-89**	4,071 (64.0%)	6,632 (63.3%)	547 (8.6%)	777 (7.4%)	44 (0.7%)	102 (1.0%)	766 (12.0%)	972 (9.3%)	934 (14.7%)	1,986 (19.0%)	6,362 (100%)	10,469 (100%)
**90+**	204 (65.2%)	465 (63.1%)	22 (7.0%)	39 (5.3%)	0	7 (0.9%)	45 (14.4%)	84 (11.4%)	42 (13.4%)	142 (19.3%)	313 (100%)	737 (100%)
**Total**	79,088 (57.2%)	98,071 (58.0%)	6,641 (4.8%)	8,258 (4.9%)	752 (0.5%)	1,184 (0.7%)	8,044 (5.8%)	8,705 (5.1%)	43,708 (31.6%)	52,907 (31.3%)	138,233 (100%)	169,125 (100%)

*LABA/ICS treatment categories percentage values were calculated for each age group and sex separately.

### Concomitant LABA and ICS users – fixed combination versus separate inhalers

Out of all “concomitant LABA and ICS users”, the proportion of patients receiving LABA and ICS only in a fixed inhaler device (“combined inhaler”) was high and increased significantly from 82.2% to 85.7% within the study period (p < 0.0001, Table 5).

**Table 5:** **“Concomitant LABA and ICS users” stratified by inhaler type for the years 2004 to 2008: “Combined Inhalers”, “Separate Inhalers”, “Combinations”**

	**Year**				
	**2004**	**2005**	**2006**	**2007**	**2008**
**Combined inhalers [fixed LABA/ICS]**	101,814 (82.2%)	126,347 (82.7%)	126,301 (82.9%)	145,464 (84.5%)	151,784 (85.7%)
**Separate inhalers [non-fixed LABA/ICS]**	15,044 (12.1%)	18,490 (12.1%)	17,767 (11.7%)	17,930 (10.4%)	16,750 (9.5%)
**Combinations**	7,015 (5.7%)	8,020 (5.2%)	8,350 (5.5%)	8,831 (5.1%)	8,625 (4.9%)

The fraction of patients receiving at least one prescription of a fixed LABA/ICS device, and in addition a separate ICS- or a non-fixed LABA/ICS-prescription (“combinations”) decreased from 5.7% to 4.9% (p < 0.0001). On the other hand, patients classified as “concomitant LABA and ICS users” receiving LABA and ICS only in separate inhalers decreased significantly from 12.1% to 9.5% (p < 0.0001, Table 5). The proportion of those patients with two separate inhalers was highest in men and women between ages of 50 and 79 years and no differences were found for sex (Table 6).

**Table 6:** Concomitant LABA and ICS users stratified by age group, sex, and inhaler type (“Combined Inhalers”, “Separate Inhalers”, “Combinations”) for the year 2008*

	**Combined Inhalers [fixed LABA/ICS)**		**Separate Inhalers [non-fixed LABA/ICS]**		**Combinations**		**All concomitant LABA/ICS categories**	
**Age group**	**Male**	**Female**	**Male**	**Female**	**Male**	**Female**	**Male**	**Female**
**0-9**	3,262 (76.9%)	1,735 (75.8%)	219 (5.2%)	135 (5.9%)	761 (17.9%)	418 (18.3%)	4,242 (100%)	2,288 (100%)
**10-19**	9,121 (88.2%)	6,208 (87.9%)	550 (5.3%)	422 (6.0%)	676 (6.5%)	430 (6.1%)	10,347 (100%)	7,060 (100%)
**20-29**	6,052 (90.9%)	7,208 (89.9%)	436 (6.5%)	525 (6.6%)	172 (2.6%)	281 (3.5%)	6,660 (100%)	8,014 (100%)
**30-39**	7,053 (88.5%)	9,034 (87.2%)	669 (8.4%)	885 (8.5%)	250 (3.1%)	444 (4.3%)	7,972 (100%)	10,363 (100%)
**40-49**	10,015 (87.1%)	14,110 (86.0%)	1,102 (9.6%)	1,515 (9.2%)	386 (3.4%)	788 (4.8%)	11,503 (100%)	16,413 (100%)
**50-59**	8,777 (85.1%)	13,239 (84.8%)	1,120 (10.9%)	1,624 (10.4%)	413 (4.0%)	748 (4.8%)	10,310 (100%)	15,611 (100%)
**60-69**	10,454 (83.7%)	14,200 (84.1%)	1,545 (12.4%)	1,906 (11.3%)	489 (3.9%)	779 (4.6%)	12,488 (100%)	16,885 (100%)
**70-79**	9,428 (83.5%)	12,121 (84.5%)	1,368 (12.1%)	1,594 (11.1%)	495 (4.4%)	625 (4.4%)	11,291 (100%)	14,340 (100%)
**80-89**	3,469 (85.2%)	5,712 (86.1%)	433 (10.6%)	650 (9.8%)	169 (4.2%)	270 (4.1%)	4,071 (100%)	6,632 (100%)
**90+**	178 (87.3%)	408 (87.7%)	17 (8.3%)	35 (7.5%)	9 (4.4%)	22 (4.7%)	204 (100%)	465 (100%)
**Total**	67,809 (85.7%)	83,975 (85.6%)	7,459 (9.4%)	9,291 (9.5%)	3,820 (4.8%)	4,805 (4.9%)	79,088 (100%)	98,071 (100%)

*Concomitant LABA/ICS treatment categories percentage values were calculated for each age group and sex separately.

## Discussion

In our study, we found a slightly improved guideline adherence in asthma patients in terms of i.) a moderate increase of concomitant LABA and ICS prescriptions (including both fixed combination drugs and separate drugs) and ii.) a slight increase of LABA/ICS fixed combination drugs between 2004 and 2008. Nevertheless, a relevant number of patients received LABA at least in one quarter without a concomitant ICS prescription (2004: 12.5%; 2008: 10.9% [including switchers, non-concomitant LABA/ICS users, LABA users without ICS]) or received non-fixed LABA/ICS treatment (2004: 12.1%; 2008: 9.5% [concomitant LABA and ICS users receiving LABA and ICS in separate inhalers]). Both issues were most frequently present in elderly men.

### LABA usage & Non-concomitant LABA/ICS usage

For formoterol we found a PPR increase between 2004 and 2007 followed by a slight decrease in 2008 whereas for salmeterol, a distinct year-by-year decrease was found between 2004 and 2008. Despite the fact, that the German drug regulatory authority has been discussed LABA in general as a drug class for which concomitant ICS prescribing is needed [7], our data cannot exclude a shift in LABA prescriptions in terms of stopping salmeterol and initiating formoterol prescribing (without ICS) in individual patients. For formoterol, an earlier onset of bronchodilative effects compared to salmeterol is well-known [20] and might explain a switch from salmeterol to formoterol. However, a lacking prescribing of ICS to asthma patients receiving formoterol has to be considered as guideline-violating prescription behaviour, too. By conducting a combined analysis of formoterol and salmeterol, we found a PPR increase between 2004 (45.2 per 10,000 persons) and 2008 (50.6 per 10,000 persons; data not shown). Furthermore, only a small proportion of patients received LABA without ICS (“switchers”, “non-concomitant LABA and ICS users” and “LABA users without ICS”) and the proportion of patients classified into these categories decreased between 2004 and 2008.

Similar to our combined analysis of formoterol and salmeterol, [19] a slight increase of the absolute number of LABA prescriptions was found in Italian asthma patients between 2006 and 2008. On the other hand, the proportion of asthmatic children receiving LABA decreased distinctly between 2001 and 2006 in a Scottish study [13]. Regarding the United States, the proportion of treatment visits with a LABA prescription without concomitant steroids decreased between 2004 and 2008 and reached less than 1% in 2008 [25]. Our data suggest a higher proportion of patients receiving LABA without ICS but methodological differences might have contributed to these discrepant results. Whereas Higashi et al. [25] calculated the proportion of visits for a particular prescription category out of all visits (including visits without LABA or ICS prescriptions) we analysed the proportion of a particular patient category based on prescriptions within one year out of all patients having at least one prescription of interest not mentioning the number of treatment visits.

### Concomitant LABA/ICS usage & Fixed LABA/ICS combination drugs

In our study, the proportion of concomitant LABA/ICS users increased from 52.0% to 57.6% between 2004 and 2008. The observed increase could be related to an increased prescribing of i.) ICS to patients receiving LABA, ii.) LABA to patients receiving ICS, or iii.) fixed LABA/ICS combinations. It is worth mentioning, that all three potential changes in prescription behaviour would be in accordance to guidelines [3]. Regarding the proportion of patients with fixed LABA/ICS combination, we found an increase between 2004 and 2008 (82.2% versus 85.7%) out of all “concomitant LABA and ICS users”. In comparison [25], LABA and ICS were prescribed concomitantly (fixed combination drug and separate compounds) in the United States in approximately 20% to 30% of asthma treatment visits. Out of these patients, 99% received a fixed combination of LABA/ICS [25].

Regarding a compound specific analysis of fixed combination drugs, we found a PPR increase of between 12.1% (salmeterol/fluticasone) and 49.8% (formoterol/beclometasone [2007–2008]) within the study period. In comparison, prescriptions of fixed salmeterol/fluticasone combination for Italian asthma patients increased between 2006 and 2008 by 45% whereas for formoterol-containing fixed combinations, a much more pronounced increase by 137% was found [19]. In a combined analysis of salmeterol- and formoterol-containing fixed combinations, more than a doubling was found for the proportion of children receiving LABA/ICS in Scotland between 2001 and 2006 [13]. On a European level [26], a 50% increase of fixed LABA/ICS combination drug prescriptions was found between 2005 and 2009.

Regarding age- and sex-stratified analyses (year 2008), the highest proportion of patients with at least one LABA prescription without concomitant ICS (combined analysis of “LABA users without ICS”, “non-concomitant LABA and ICS users”, “switchers”) was found in elderly men. The “lack” of ICS prescribing in these elderly men receiving LABA might be related to a relevant COPD drug burden (i.e. asthma-COPD overlap syndrome). However, not prescribing ICS to ACOS patients on a regular base might be reasonable regarding most recent data [27].

### Gap between guidelines/regulatory decisions and clinical practice

The knowledge gap between research and clinical practice is a well-known problem and a wide range of interventions has been evaluated. To sum up the available evidence, multifaceted, interactive approaches (e.g. audits and feedback, workshops, reminders) seems to be more effective than passive, single interventions (e.g. educational materials [28,29]). For asthma, several strategies for improving knowledge translation have been evaluated. For example, a multiple level intervention was conducted in Canada including the individual patient, the practice, and the health system level. By implementing six guideline-based care elements including e.g. spirometry measurement, asthma controller therapy, and self-management action plans, a significant improvement of relevant clinical endpoints (e.g. reduced number of urgent / emergent healthcare visits [30]) was found. Furthermore, the proportion of patients receiving LABA/ICS combination therapy increased, but unfortunately, results for LABA monotherapy were not reported in this study. Most recently, electronic tools were examined to promote knowledge translation at physician and patient level, but further studies are needed to clarify the impact of these approaches [31,32] in particular regarding their impact on improving guideline-adherent prescription behaviour.

In Germany, a nationwide disease management program (DMP) for patients with asthma was implemented in 2006 including e.g. regular visits, individual action plans, and regular feedback to coordinating general practitioners [33]. By analysing annual trends of asthma treatment for patients included in this DMP between 2006 and 2010, a guideline-adherent prescribing was found in the majority of patients. Nevertheless, a small but slightly increasing proportion of patients has received LABA monotherapy (2006: 2.1%, 2010: 3.5%). As already discussed for our study, this finding could be related to a concomitant diagnosis of COPD which was present in 2.8% to 5.2% of DMP patients [33].

Regarding the impact of regulatory actions on prescribing behaviour in daily practice, only a few well-conducted studies are available. In a systematic review, no final conclusion regarding the impact of safety-related regulatory actions could be made due to inadequate study designs and heterogeneity in analyses and outcome measures [34]. In particular, confounding factors are of outstanding importance but difficult to adjust for hampering a valid estimate of the actual impact of a particular regulatory action. By comparing the impact of different information included in a “Dear doctor” letter, “simple” information (dose limit) has been considered by physicians more frequently compared to “complex” information (QT prolongation due to drug-drug interactions) [35]. Taken into account the more or less “simple” message of asthma guidelines and SMART-related regulatory actions, a guideline adherent prescribing of LABA and ICS seems achievable. Due to confounding factors (e.g. disease management program for asthma patients) and the complexity of treatment decision making, we were unable to quantify the actual impact of the SMART trial publication and/or SMART-related drug regulatory actions on prescribing behaviour.

### Strengths & limitations

Our study has several strengths worth noting. First, the database covers a population of about 10.5 million people and represents 85% of the inhabitants of Bavarian (the largest federal state of Germany). In addition, we were able to perform detailed analyses focusing on the presence or absence of concomitant ICS prescriptions in asthma patients receiving LABA. Furthermore, due to a 5-year period, we could analyse trends in PPRs as well as in LABA- and ICS-related prescription behaviour after the SMART trial. Despite these strengths, there are also several limitations regarding our study. Firstly, our analysis was limited to the years 2004 to 2008 and more recent changes in prescription behaviour should be taken into account. By comparing nationwide drug prescription data for the years 2008 and 2013 (irrespective of indication), for salmeterol a 72.5% decrease (2008: 9.1 Mio. DDD, 2013: 2.5 Mio. DDD), for formoterol a 22.2% increase (2008: 99.1 Mio. DDD, 2013: 121.1 Mio. DDD), for ICS a slight decrease of 4.0% (2008: 167.6 Mio. DDD, 2013: 160.9 Mio. DDD), and for fixed combinations of LABA/ICS a distinct increase of 28.7% (2008: 255.8 Mio. DDD, 2013: 329.2 Mio. DDD) were found [36,37]. Since our main aim was to conduct a comprehensive and more detailed analysis of LABA- and ICS-prescribing in asthma patients after the SMART trial, we decided to use a period covering most factors potentially influencing drug prescribing including publication and dissemination of SMART results and SMART-related drug regulatory actions. Secondly, the prescriptions are documented on a quarterly basis in the database meaning that all LABA and ICS prescriptions were counted as concomitant if they occurred in the same quarter irrespective of the actual prescription date. Hence, the proportion of concomitant LABA/ICS users is likely to be overestimated in our study. Thirdly, only patients with compulsory insurance are covered in our analyses and thus, by excluding patients with a private health insurance, a socioeconomic bias on our study results cannot be excluded. Fourthly, this study covers a Southern region of Germany and hence, generalizability of results to other German regions or to other countries is limited as reported for several other studies, too [38,39]. However, by taking into account methodological differences, PPRs for LABA, ICS, and fixed combination of LABA/ICS found in our study showed a similar pattern and trend compared to national drug consumption data [36,40]. Fifthly, by using prescription data only, prescribing quality can only roughly assessed due to missing individual data important for a clinical decision (e.g. lacking lung function parameter, detailed clinical history) and a substantial proportion of patients potentially given an incorrect diagnosis (i.e. asthma instead of COPD) [41,42].

## Conclusions

By analysing prescription data of a German population covering 10.5 million subjects, we found a slightly increased guideline adherence between 2004 and 2008. In elderly men, the proportion of patients receiving LABA and ICS non-concomitantly was highest but might be reasonable taking into account a concomitant diagnosis of COPD (i.e. ACOS). Due to the complexity of factors influencing prescription behaviour (e.g. guidelines, disease management programs), we were not able to quantify the actual impact of the publication or dissemination of the SMART trial results and/or related drug regulatory actions. Further studies are needed to analyse in detail the impact of “milestone” trials and related drug regulatory actions on real-life prescription behaviour.

## Additional file

Additional file 1:
**Table S1.** Annual period prevalence rates per 10,000 persons stratified by compound between 2004 and 2008. **Table S2.** Number and proportion of patients with an additional COPD diagnosis for the year 2008.

## Abbreviations

ATC: Anatomical Therapeutic Chemical classification system
ICD-10-GM: International Classification of Diseases and Related Health Problems terminology (German Modification)
ICS: Inhaled corticosteriod
LABA: Long-acting beta-2-agonist
PPR: Period prevalence rate
SD: Standard deviation
SMART: Salmeterol Multicentre Asthma Research Trial
