# Supplementary material for: Prescribing of long-acting beta-2-agonists/inhaled corticosteroids after the SMART trial
Source: BMC Pulm Med. 2015 May 6;15:55. doi: 10.1186/s12890-015-0051-x (PMC4428117; doi:10.1186/s12890-015-0051-x)
Supplement: Additional file 1: — Table S1. Annual period prevalence rates per 10,000 persons stratified by compound between 2004 and 2008. Table S2. Number and proportion of patients with an additional COPD diagnosis for the year 2008. [file 12890_2015_51_MOESM1_ESM.docx]

Additional files

Additional Table S1: Annual period prevalence rates per 10,000 persons stratified by compound between 2004 and 2008

|  |  | Annual period prevalence rates per 10,000 persons | | | | |
| --- | --- | --- | --- | --- | --- | --- |
| Compound | ATC | 2004 | 2005 | 2006 | 2007 | 2008 |
| Salmeterol | R03AC12 | 9.8 | 8.6 | 6.7 | 5.6 | 4.6 |
| Formoterol | R03AC13 | 35.8 | 44.6 | 46.3 | 47.1 | 46.2 |
| Salmeterol / fluticasone | R03AK06 | 62.1 | 73.1 | 70.5 | 70.7 | 69.6 |
| Formoterol / beclometasone | R03AK27 | - | - | 2.4* | 18.2 | 27.3 |
| Formoterol / budesonide | R03AK28 | 49.7 | 63.4 | 64.2 | 69.2 | 67.6 |
| Beclometasone | R03BA01 | 28.6 | 29.6 | 32.1 | 33.7 | 37.4 |
| Budesonide | R03BA02 | 75.6 | 82.6 | 86.9 | 90.6 | 87.7 |
| Fluticasone | R03BA05 | 15.2 | 15.3 | 14.2 | 12.7 | 11.5 |
| Mometasone | R03BA07 | 1.8 | 1.1 | 0.4 | 0.2 | 0.1 |
| Ciclesonide | R03BA08 | - | 6.2** | 11.0 | 13.1 | 4.1 |
| * Drug approval in 07/2006; ** Drug approval in 01/2005 | | | | | | |

Additional Table S2: Number and proportion of patients with an additional COPD diagnosis for the year 2008.

| **Age group** | **Concomitant LABA and ICS users** | | **Switchers** | | **Non-concomitant LABA and ICS users** | | **LABA users without ICS** | | **ICS users without LABA** | |
| --- | --- | --- | --- | --- | --- | --- | --- | --- | --- | --- |
|  | **Male** | **Female** | **Male** | **Female** | **Male** | **Female** | **Male** | **Female** | **Male** | **Female** |
| **40-49** | 2,693 (23.4%) | 3,432 (20.9%) | 291 (36.8%) | 322 (28.5%) | 35 (36.8%) | 38 (21.2%) | 289 (32.0%) | 301 (24.6%) | 794 (16.2%) | 1,200 (14.6%) |
| **50-59** | 4,500 (43.6%) | 5,756 (36.9%) | 601 (60.1%) | 761 (50.9%) | 60 (53.6%) | 80 (38.3%) | 651 (57.8%) | 671 (46.7%) | 1,102 (27.8%) | 1,676 (23.1%) |
| **60-69** | 7,381 (59.1%) | 7,816 (46.3%) | 1,236 (74.5%) | 1,178 (61.3%) | 98 (66.2%) | 130 (53.7%) | 1,221 (68.6%) | 1,033 (57.6%) | 1,608 (38.2%) | 2,257 (30.2%) |
| **70-79** | 7,699 (68.2%) | 7,826 (54.6%) | 1,350 (80.7%) | 1,192 (67.0%) | 124 (68.9%) | 152 (66.4%) | 1,372 (73.4%) | 1,104 (62.4%) | 1,576 (47.8%) | 2,205 (40.5%) |
| **80-89** | 2,998 (73.6%) | 3,966 (59.8%) | 431 (78.8%) | 562 (72.3%) | 35 (79.5%) | 63 (61.8%) | 597 (77.9%) | 629 (64.7%) | 564 (60.4%) | 895 (45.1%) |
| **90+** | 137 (67.2%) | 247 (53.1%) | 15 (68.2%) | 30 (76.9%) | 0 | 5 (71.4%) | 29 (64.4%) | 52 (61.9%) | 24 (57.1%) | 64 (45.1%) |
